# Classification of frequency response areas in the inferior colliculus reveals continua not discrete classes

**DOI:** 10.1113/jphysiol.2013.255943

**Published:** 2013-06-10

**Authors:** Alan R Palmer, Trevor M Shackleton, Christian J Sumner, Oliver Zobay, Adrian Rees

**Affiliations:** 1 Medical Research Council Institute of Hearing Research, University Park Nottingham NG7 2RD, UK; 2 Institute of Neuroscience, Faculty of Medical Sciences, Newcastle University Newcastle upon Tyne NE2 4HH, UK

## Abstract

A differential response to sound frequency is a fundamental property of auditory neurons. Frequency analysis in the cochlea gives rise to V-shaped tuning functions in auditory nerve fibres, but by the level of the inferior colliculus (IC), the midbrain nucleus of the auditory pathway, neuronal receptive fields display diverse shapes that reflect the interplay of excitation and inhibition. The origin and nature of these frequency receptive field types is still open to question. One proposed hypothesis is that the frequency response class of any given neuron in the IC is predominantly inherited from one of three major afferent pathways projecting to the IC, giving rise to three distinct receptive field classes. Here, we applied subjective classification, principal component analysis, cluster analysis, and other objective statistical measures, to a large population (2826) of frequency response areas from single neurons recorded in the IC of the anaesthetised guinea pig. Subjectively, we recognised seven frequency response classes (V-shaped, non-monotonic Vs, narrow, closed, tilt down, tilt up and double-peaked), that were represented at all frequencies. We could identify similar classes using our objective classification tools. Importantly, however, many neurons exhibited properties intermediate between these classes, and none of the objective methods used here showed evidence of discrete response classes. Thus receptive field shapes in the IC form continua rather than discrete classes, a finding consistent with the integration of afferent inputs in the generation of frequency response areas. The frequency disposition of inhibition in the response areas of some neurons suggests that across-frequency inputs originating at or below the level of the IC are involved in their generation.

**Key points**

- Neurons in the auditory midbrain, the inferior colliculus, are selectively sensitive to combinations of sound frequency and level as illustrated by their frequency/level receptive fields. Different receptive field shapes have been described, but we do not know if these represent discrete classes reflecting afferent inputs from individual sources, or a more complex pattern of integration.
- In this study we used objective methods to analyse the receptive fields of over 2000 neurons in the guinea pig inferior colliculus.
- Subjectively we identified seven different receptive field classes, but objectively these classes formed continua with many neurons having receptive field shapes intermediate to these extremes.
- These findings are consistent with neurons receiving inhibitory inputs of different strength and frequency disposition but not consistent with neurons reflecting inputs only from individual brainstem nuclei.
- These results are important for understanding the functional organisation of the inferior colliculus and its role in auditory processing.

## Introduction

Delineating the organisation of sensory receptive fields has been an important goal in sensory neuroscience since the pioneering studies of Hubel & Wiesel (1962). In the auditory system, receptive fields are often defined in the spectral domain. The spectral analysis of sound, so fundamental to hearing, begins in the cochlea where maximal vibration of the basilar membrane varies systematically with frequency along its length (von Bekesy, 1949; Robles & Ruggero, 2001), resulting in auditory nerve fibres having a narrow V-shaped frequency tuning function often accompanied by a low frequency tail (Kiang *et al.* 1965). This tuning is a defining feature characterising auditory neurons and is often quantified as the response to different frequencies as a function of sound level: the frequency response area. Such frequency response areas have been described at all levels of the auditory pathway, and in the inferior colliculus (IC), the midbrain nucleus of the auditory pathway, they have been described in several species (Ehret & Merzenich, 1988; Casseday & Covey, 1992; Yang *et al.* 1992; Ramachandran *et al.* 1999; Egorova *et al.* 2001; LeBeau *et al.* 2001; Hernández *et al.* 2005; Alkhatib *et al.* 2006). Although some IC neurons have V-shaped response areas, similar to those of primary auditory nerve fibres, others have substantially different shapes indicative of the interplay of excitation and inhibition in shaping these receptive fields. Discovering how frequency response areas of neurons in the IC are generated is important in understanding the organisation of the IC and its role in auditory processing, since it is an almost obligatory site of termination of inputs from nearly all (>10) lower brainstem nuclei and receives descending connections from the thalamo-cortical centres (Oliver & Shneiderman, 1991; Malmierca & Hackett, 2010).

Anatomical studies show that afferent inputs from key brainstem nuclei such as the cochlear nuclei, the superior olivary complex and the lateral lemniscus are to an extent differentially distributed within the IC. This is true between the major subdivisions and within the subdivisions at the level of microcircuits, in what have been termed synaptic domains (Brunso-Bechtold *et al.* 1981; Oliver & Huerta, 1992). Nevertheless, there is considerable overlap between the terminals of afferent inputs from different sources and hence high potential for connections between synaptic domains (Cant, 2005; Schofield, 2005).

In an influential study of the IC of the decerebrate cat, Ramachandran *et al.* (1999) proposed the existence of three distinct response area types (V, I and O) which, on the basis of response area shape and their distribution with frequency, they argued could be accounted for by input from three specific brainstem sources, the medial and lateral superior olive and the dorsal cochlear nucleus, respectively (Davis *et al.* 1999; Ramachandran *et al.* 1999). Evidence for the inheritance of the type O from the dorsal cochlear nucleus (DCN) was supported by inactivation experiments (Davis *et al.* 1999). On the other hand, studies combining electrophysiological recording in the IC with microiontophoresis of inhibitory antagonists have emphasised the role of inhibition operating within the IC itself, either from afferent inputs or from IC interneurons, in generating different response types (Vater *et al.* 1992; Yang *et al.* 1992; Palombi & Caspary, 1996; LeBeau *et al.* 2001).

If response areas in the IC are dominated by relatively pure sources of input as Ramachandran *et al.* (1999) proposed, one might predict that they should readily segregate into a number of discrete classes. One of the difficulties in addressing such issues in the IC is the relatively small number of units representing each response area type obtained in many studies, particularly where relatively low yield techniques like iontophoresis or inactivation are involved. Here we test the hypothesis that IC response areas fall into a small number of classes by applying cluster analysis and other objective quantitative analyses to a large sample, over 2800, of response areas from neurons in the IC of the anaesthetised guinea-pig. Although our analysis shows that descriptive classes can be defined, these groupings are not discrete, but rather occur as a series of continua. These data are consistent with the notion that the frequency responses of IC neurons reflect widespread synaptic integration.

## Methods

### Ethical approval

The experiments described in this study were performed under the terms and conditions of licences issued by the UK Home Office under the Animals (Scientific Procedures) Act 1986, project licence number 4003049, and the approval of the ethical review committee of the University of Nottingham.

### Preparation and anaesthesia

The data we report were collected over a period of more than 20 years in 359 experiments on the inferior colliculus of anaesthetised, mature, pigmented guinea pigs. The frequency response area (FRA) is measured in our laboratories as a routine part of characterising the sensitivities of central auditory neurons so allowing other analyses to be optimised for the single neuron under study. The other data gathered in such experiments have provided the basis for a large number of publications detailing different aspects of neural activity in the inferior colliculus.

The presence and type of anaesthetic may make a material difference to the balance of excitation and inhibition that is observed in frequency response areas (see Evans & Nelson, 1973; Young & Brownell, 1976; Rhode & Kettner, 1987). Initially we used a neuroleptic technique (*n*= 34, 323 units) developed for the guinea pig (see Evans, 1979; Caird *et al.* 1991 for details). This included pentobarbitone with further analgesia provided by maintenance doses of phenoperidine. Pentobarbitone has been shown to affect inhibition in the auditory pathway (Evans & Nelson, 1973; Rhode & Kettner, 1987) and we subsequently replaced pentobarbitone with urethane, while still using phenoperidine to provide the additional analgesia (see Jiang *et al.* 1996 for details: *n*= 177, 1652 units). When phenoperidine became unavailable a small number of animals (6) were anaesthetised with Hypnorm (Janssen) combined with medazolam (Hypnoval; Roche), before we adopted our present technique in which urethane is supplemented with Hypnorm (see McAlpine & Palmer, 2002 for details: *n*= 140, 818 units). This has proved to be an effective regime for all levels of the auditory pathway of the guinea pig from auditory nerve to cortex, and urethane has been shown to produce anaesthesia with minimal effects on inhibition in cortical neurons (Sceniak & MacIver, 2006). In all cases atropine sulphate (0.06 mg kg^−1^ s.c.) was administered to reduce bronchial secretions. Dosage regimes for the three major protocols were as follows:

1. *Neuroleptic:* sodium pentobarbitone (30 mg kg^−1^ i.p.), phenoperidine (1 mg kg^−1^ i.m.), and droperidol (4 mg kg^−1^ i.p.; Evans, 1979). Supplementary doses of phenoperidine (1 mg kg^−1^) and pentobarbitone (6 mg kg^−1^) were given as required as indicated by the pedal withdrawal reflex.
2. *Urethane plus phenoperidine*: urethane (0.9–1.3 g kg^−1^ in 20% solution i.p.; Sigma) and phenoperidine (1 mg kg^−1^ i.m.). Supplementary doses of phenoperidine (1 mg kg^−1^) were given as required as indicated by the pedal withdrawal reflex.
3. *Urethane plus Hypnorm*: urethane (0.9–1.3 g kg^−1^ in a 20% solution i.p., Sigma) and fentanyl/fluanisone (Hypnorm: 0.2 ml i.m., fentanyl citrate, 0.315 mg ml^−1^+ fluanisone, 10 mg ml^−1^; formerly Janssen, currently VetaPharma). Supplementary doses of fentanyl/fluanisone (0.2 ml i.m.) were given as required as indicated by the pedal withdrawal reflex.

The end-point of an experiment, usually 12–20 h after induction, was indicated by completion of sampling, loss of cochlea sensitivity or the death of the animal under anaesthesia. In the first two cases, the animal was killed by an overdose of sodium pentobarbitone. In some experiments, under deep terminal anaesthesia, the animal was perfused through the heart and the brain removed for histological examination to recover electrolytic lesions that were used to confirm that recordings were from the central nucleus of the IC.

### Electrophysiological recording

Details of the surgical preparation can be found in Rees & Palmer (1988). Briefly, all animals were tracheotomised and core temperature was maintained at 38°C with a heating blanket, monitored by a rectal probe. In some cases, the animal was artificially ventilated. The animal was placed inside a sound-attenuating room in a stereotaxic frame with the ear-bars replaced by hollow plastic speculae. Pressure equalisation within the middle ear was achieved by narrow polythene tubes sealed into small holes in the bullae. A craniotomy was performed in the skull above the IC and electrodes were introduced via a dorso-ventral approach through the overlying cerebral cortex. Responses from well isolated single neurons were measured using single tungsten-in-glass microelectrodes. These were originally commercially available (Neurolog NL02), but when these were discontinued they were manufactured in-house (Bullock *et al.* 1988).

We used the conventional on-line physiological criteria originally proposed by Rose *et al.* (1963) and subsequently used by many researchers to determine that our recordings were from the central nucleus of the inferior colliculus. These include a short latency, brisk and often sustained response to single tone bursts, appropriate sensitivity to binaural cues (interaural time delay and interaural level difference), electrode depth below the cortical surface and a dorso-ventral tonotopic progression of characteristic frequency (CF; Rose *et al.* 1959). In some experiments histological verification of the recordings site was achieved using electrolytic lesions in sections stained with cresyl violet and in a few the cell was juxtacellularly filled with biocytin and computer reconstructed (Wallace *et al.* 2012). We are confident that the vast majority of our recordings were from the central nucleus, but we cannot state that unequivocally in every case.

### Sound stimulation

Over the period of data collection we changed computer several times and sound system once. Both closed sound systems that we used were flat (±10 dB) to 20 kHz with a maximum output of about 100 dB SPL (see for examples: Winter & Palmer, 1990; Palmer *et al.* 1996). Sound levels near the eardrum were routinely measured in every experiment and converted to dB SPL (Sound Pressure Level: dB re. 20 micro Pascal) using a calibrated 1 mm probe tube attached to a half-inch Brüel and Kjaer condenser microphone (type 4134). We monitored minimum thresholds measured before and after changing the sound systems and found no differences. However, all frequency response areas in this paper are plotted on a decibel scale referenced to the maximum system output, i.e. attenuation in decibels below approximately 100 dB SPL. All classifications and other measurements are based on levels with respect to the neurons’ thresholds and CFs.

### Measuring the frequency response area

**Figure 1:**
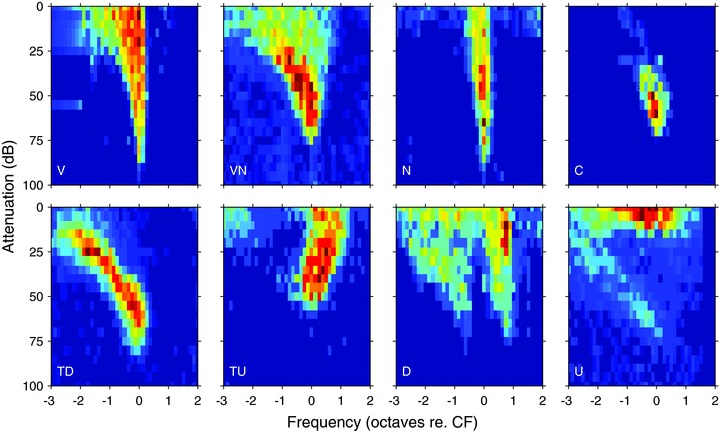
Exemplars of the subjective frequency response area classes in the IC on an octave frequency scale relative to CF Each response area was normalised by dividing by the maximum firing rate within the area. The CFs of the units were 14.8, 1.2, 8.0, 0.69, 1.5, 0.55, 5.0 and 0.25 kHz reading from top left to bottom right.

**Figure 2:**
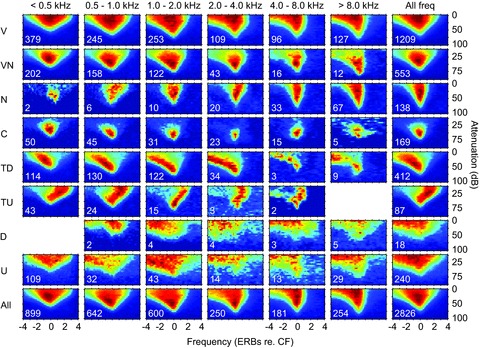
Subjective classes of response area plotted as a function of response area type (rows) and CF in octave bands (columns) See text for details of the normalisation process. Each plot is the mean of all frequency response areas of that type in that frequency range. The number of neurons included is shown at the bottom left corner of each plot. The final row shows the average of all units irrespective of response area type including D and unclassified. The final column shows the mean of the response type irrespective of CF.

The method we used to determine the frequency response area has not changed materially over the years, enabling us to combine data from the archives. On isolating a neuron its CF and threshold at CF were determined using audio-visual criteria. The frequency response area was then compiled from 50 ms tones presented in a pseudo-random sequence at 4 s^−1^ over a frequency range of three or four octaves below, to two octaves above, the unit's CF in 1/8th octave steps, and over a 100 dB range of sound levels in 5 dB steps. The number of spikes elicited by each frequency and level combination was displayed during the data collection as a block at the appropriate frequency and level position which was coded proportionally to the spike count using a colour temperature scale (see Figs 1 and 2 for examples).

**Figure 14:**
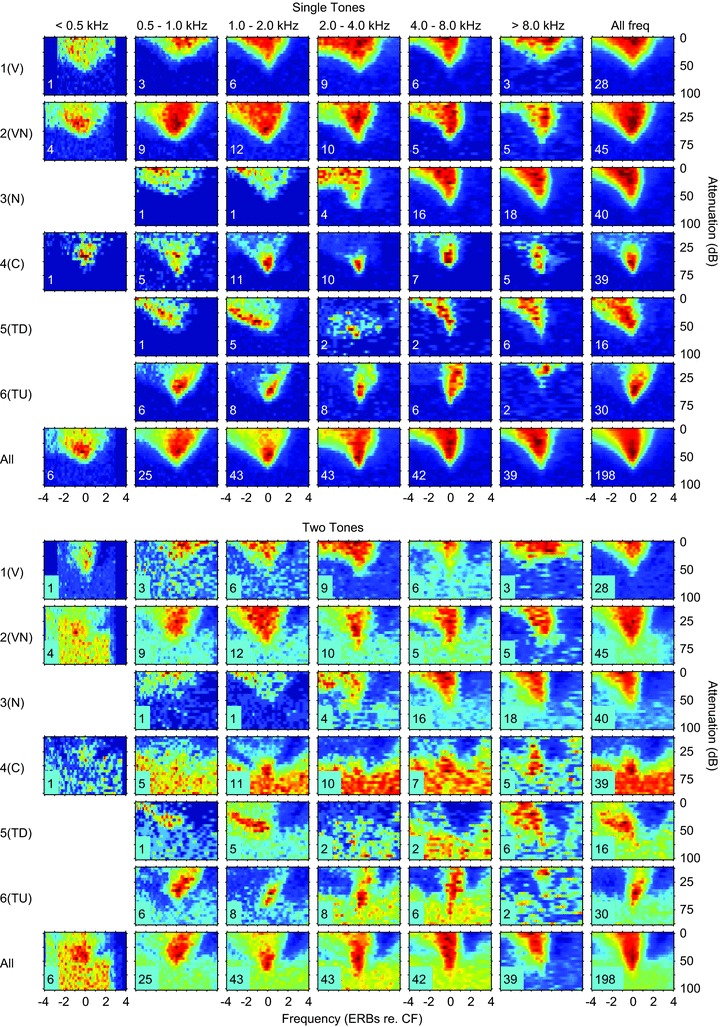
Comparison of single-tone response areas and those obtained with two tones: a single tone at 10 dB above threshold at CF and a roving tone at the level and frequency shown by the axes Top: averaged frequency response areas as a function of the CF. All response areas in the frequency range shown at the top were normalised and then averaged for the different classes. The normalised spike counts are shown on a temperature scale with dark blue as 0% and dark red as 100%. The numbers on each response area show the number of units averaged. Below: corresponding averaged two-tone response areas for the same units: see text for details.

In some cases, the spontaneous rate of a neuron was sufficiently high that inhibitory areas could be revealed by the inhibition of spontaneous activity obtained by the presentation of single tones. Where spontaneous activity was low or absent, inhibitory contributions to the response area could be revealed by measuring the response area in the presence of a CF tone that was just sufficiently supra-threshold to evoke spiking activity. We often measured such two-tone response areas in later experiments with the second tone at 10 dB suprathreshold (see Fig. 14 for examples).

In the first 29 animals we stimulated only the contralateral ear, while in all of the remaining 330 experiments stimulation was binaural. Activity in the inferior colliculus is dominated by contralateral responses and we found no major differences in the distribution of response area types between the contralateral or diotically stimulated animals.

With no obvious differences across these various experimental protocols, we pooled all of the data for the classification analyses in this paper giving a total of 2826 that were sufficiently complete to allow assessment of their shape.

### Classification of frequency response areas

Response areas were initially subjectively classified using the traditional technique of visual appearance. We also developed an automatic classification algorithm, partly guided by this visual classification. The response areas were summarised either through principal component analysis (PCA) or via extraction of a selection of parameters describing aspects of the response areas. The response areas were then classified using cluster analysis. The actual number of clusters requested and the number of parameters used were determined by analysis of cluster validity indices (CVIs: Xu & Wunsch, 2009, see below and results for further details). Further measurements of bandwidth and *Q*-factor were made using a separate technique and plotted against CF.

#### Subjective classification

Response areas plotted on the usual logarithmic scale were classified by two of us (A.R.P. and A.R.) independently in terms of the gross shape of the receptive field across level: broadening or remaining narrow; the degree of non-monotonicity and movement of the centre of masses. These were then evaluated (together with T.M.S.) and a final classification developed. Seven distinct response area types were defined, as discussed in the first section of Results (see Fig. 1): (1) V (V-shaped, like auditory nerve), (2) VN (V-non-monotonic), (3) N (narrow), (4) C (closed), (5) TD (tilt down), (6) TU (tilt up), and (7) D (double-peaked) having two peaks of sensitivity. Response areas which were not sufficiently reliable to allow classification, or did not fit into any of the seven classes, were classed as U (unclassified).

#### Response area normalisation

It is well known that the width of tuning curves plotted on a logarithmic scale changes with CF. This *change* occurs at the level of the basilar membrane, so it is not an emergent property at the IC and may mask more subtle emergent properties. We therefore normalised the response areas to a function based on the equivalent rectangular bandwidth (ERB)-rate frequency scale (Moore & Glasberg, 1983) derived from a power function fit to the equivalent rectangular bandwidth (ERB = 0.34 × CF^0.73^) calculated for V-type units described in the Results to remove the CF dependence. The audio-visually defined CF was checked using the method described in ‘Extraction of measures of tuning from the frequency response areas’ below and adjusted if necessary. Response areas were then linearly interpolated (using MATLAB ‘interp2’ function) onto an ERB-rate scale from 4 ERBs below CF to 4 ERBs above in steps of 0.1 ERBs, and over a sound level range of 100 dB in 5 dB steps. The firing rates were normalised by dividing all values by the maximum firing rate within the response area.

#### Principal component analysis (PCA)

The entire frequency response areas of all units were analysed using PCA (MATLAB function ‘princomp’). This takes into account all of the features of the frequency response area. Parallel analysis (Horne, 1965) was used to determine which principal components were significant. Parallel analysis attempts to determine the number of ‘true’ factors generating a noisy data set. This is achieved by comparing the ranked eigenvalues of the observed data covariance or correlation matrix to the distribution of the corresponding eigenvalues for completely uncorrelated data.

#### Descriptive parameter extraction

It would have been optimal if the clustering structure were derivable from the raw data. However, this could not be expected *a priori*, since the raw frequency response areas contain considerable information that is irrelevant for our purposes. Therefore, in order to guide the cluster analysis, it seemed sensible to base clustering on extracted parameters that focused on features of interest. We extracted a large set of parameters (18: see Table 1) that quantified the shape and magnitude of the responses as a function of level and frequency (see below for details of the parameters).

**Table 1:** Parameters extracted from the response areas

Parameter number	Description	Units
1	Variation of the BF as a function of sound level	ERBs dB^−1^
2	Variation of the width of the response with sound level	ERBs dB^−1^
3	Average width of the response area (based on standard deviation of isolevel functions)	ERBs
4	Normalised maximum firing rate below CF	
5	Normalised maximum firing at CF	
6	Normalised maximum firing above CF	
7	Sound level relative to threshold at which maximum firing rate occurs below CF	dB
8	Sound level relative to threshold at which maximum firing rate occurs at CF	dB
9	Sound level relative to threshold at which maximum firing rate occurs above CF	dB
10	Slope of the normalised rate level function between threshold and peak below CF	dB^−1^
11	Slope of the normalised rate level function between threshold and peak at CF	dB^−1^
12	Slope of the normalised rate level function between threshold and peak above CF	dB^−1^
13	Monotonicity below CF: ratio of the maximum of the rate level function to the rate at the highest sound level at CF	
14	Monotonicity at CF: ratio of the maximum of the rate level function to the rate at the highest sound level below CF	
15	Monotonicity above CF: ratio of the maximum of the rate level function to the rate at the highest sound level above CF	
16	Difference between the normalised maximum firing rate at and above CF	
17	Difference between the normalised maximum firing rate at and below CF	
18	Difference between the normalised maximum firing rate above and below CF	

The full analysis for the extraction of the parameters used in the classification was as follows. The maximum firing rate in the whole response area was used to normalise the response areas. An isolevel response function was formed across frequency at each sound level from 5 dB below threshold to 60 dB above. Peaks and troughs in this function were found, and the peak with the largest area was determined. The centroid of this peak and its standard deviation were calculated in ERBs. The centroid of the peak was defined as the best frequency (BF, frequency of largest peak in an isolevel response function) at this level. The centroid position and standard deviation were plotted as a function of sound level and the averages and slopes across level were calculated. Rate-level functions (RLFs) at CF, at 0.75 ERBs above and 1.0 ERB below CF were constructed, from which the ratios of the maximum firing rate to the firing rate at the highest sound level were computed as measures of monotonicity. We calculated the level relative to threshold at which the maximum firing rate in the RLF occurred and the slope of the RLF between threshold and maximum. We also calculated the ratios between the maxima of the RLFs at different frequencies. To ensure that each of the parameters had equal weight in the clustering we standardised the values (subtracted the mean and divided by the standard deviation) to remove variations in the absolute values that could be taken by the different parameters.

#### Automatic classification using cluster analysis

Classification was achieved by a *k*-means clustering algorithm using a squared Euclidean distance measure (MATLAB ‘*k*means’ function) with random cluster seeds and 100 repeats to avoid local minima. The clustering algorithm was instructed to search for between two and nine clusters using various sets of parameters as described below. When clustering principal components only the significant components were used. When clustering descriptive parameters the number of parameters used was varied to optimise the CVIs.

#### Calculation of cluster validity indices (CVIs)

To assess the internal validity of any emergent clustering, we calculated CVIs (Xu & Wunsch, 2009). These are intended to answer the crucial question of whether the groups form actual clusters, i.e. whether the objects within a group are similar to each other, but different from members of other groups (in terms of the distance measure). Several different CVIs have been proposed in the literature based on comparing a characteristic intra-cluster size to some measure of between-cluster distance. To avoid over-reliance on any one of these measures we tested the data using six different well-established CVIs: Dunn index (Dunn, 1973); Caliński–Harabasz index (Caliński & Harabasz, 1974); I index (Maulik & Bandyopadhyay, 2002); inverted C index (Hubert & Levin, 1976); inverted Davies–Bouldin index (Davies & Bouldin, 1979); and silhouette (Rousseeuw, 1987). The C index and Davies–Bouldin index indicate best clustering when they are at a minimum, so the reciprocal was calculated so that all indices indicated best clustering with a maximum. For the descriptive parameter-based clustering, when we varied the number of parameters in order to avoid any biases based on parameter selection, we based the CVIs on the full 18-dimensional space defined by all parameters even if some were not used.

#### Reduction of number of descriptive parameters

In calculating clusters based on PCA we used all the principal components that were significant. However, we were aware that the set of descriptive parameters chosen was to some extent arbitrary and attempted to determine which parameters best aided classification. We attempted several techniques for reducing the number of parameters used in classification to a more manageable number; however, we finalised on an automatic selection technique. We calculated the CVIs for *all* combinations of a number of parameters. The CVIs of the combinations were then ranked for that number of parameters from highest to lowest value for each CVI individually; finally the median rank across CVIs was calculated and the lowest (i.e. best) rank chosen. This method found the combination of parameters which, assessed by the quantitative criterion of CVIs, gave the most distinct clusters for that number of parameters. It was not possible to permute more than four parameters due to computational time and memory constraints so this method was only used directly for two, three or four parameters. To find solutions with more than four parameters (6, 8, 9, 12 and 16), the chosen parameters were then included in the parameter list and a further two, three or four chosen by the same technique; this was repeated to obtain solutions with up to 16 parameters (not all possible combinations were obtained). Thus, for example, it was possible to obtain results for 6 parameters by a variety of routes (i.e. 2 + 4, 4 + 2, 2 + 2 + 2 or 3 + 3). This is a form of sequential feature selection which avoids some, but not all, of the problems of converging to a local minimum. Alternatively, we also determined parameter sets formed by removing 2, 3, 4 or 8 (4 followed by another 4) parameters from the full set of parameters to obtain solutions with 10, 14, 15 and 16 parameters. A few (16/2826) units needed to be eliminated from the analysis at this point because it was not possible to calculate the full feature set for them. After this procedure 2810 units remained in the data set.

#### Presentation of clusters based on descriptive parameters

The clustering algorithm produces clusters in random order, so to ease interpretation we ordered the clusters obtained from the descriptive parameters according to their similarity to the subjective clusters obtained earlier. The centroid of each subjective class and each objective cluster in the *n*-dimensional space defined by the parameter set used was first determined. The Euclidean distance between the centroids of each of the subjective classes and each of the objective clusters was then calculated. The objective cluster was assigned to the subjective class to which it was closest. If two objective clusters were closer to a single subjective class than any others, the closer cluster was assigned and the further assigned to the next best match. Since all objective clusters had to be assigned to a subjective group the match might not be very good for the last cluster assigned. Thus, the assignment may just indicate that no other objective cluster is closer to the subjective class than the one that is assigned, not that it is particularly close. It should be noted that this algorithm has no effect on the objective clusters obtained, just on how they are labelled. To indicate that the assignments are tentative the objective clusters are labelled as 1(V), 2(VN), 3(N), 4(C), 5(TD) and 6(TU), corresponding to the subjective classes V, VN, N, C, TD and TU, respectively, described above.

### Extraction of measures of tuning from the frequency response areas

A set of tuning parameters was extracted from each response area to permit calculation of various measures of bandwidth and quality factor (*Q*) for comparison with similar data from auditory nerve and cochlear nucleus. Frequency response areas measured with single tone presentations were often quite noisy and to obtain a reliable estimate of the tuning parameters automatically it was necessary to pre-process the response areas. The technique adopted was adapted from an automatic method used in other research (Sumner & Palmer, 2012) so is distinct from that described earlier, although the pre-processing has a number of similar features.

The frequency response areas were first up-sampled by a factor of two in frequency and sound level (using MATLAB ‘interp2’ function). The data were then smoothed (using the MATLAB function ‘filter2’) using a two-dimensional filter kernel derived from the matrix multiplication of a triangular window with a 2.5 dB ‘half-width’ ([0.5 1 0.5] across the interpolated sound levels) and a Gaussian with standard deviation 1/16th octave across frequency. The resulting filter kernel was normalised so that the sum of all elements was one. To minimise edge effects when the window overlapped the edge of the response area, the response areas were padded with extra values (the median of the three nearest neighbours) around the edge.

The mean and standard deviation of the unit's spontaneous rate was calculated from the spike counts at the minimum sound level across all frequencies, which were below the unit's audio-visual threshold. An automatic threshold was defined for each frequency step as the sound level at which the number of spikes elicited met the following criteria: the measured rate was equal to the greater of (i) the mean spontaneous rate plus 4 standard deviations of the spontaneous rate or (ii) the mean spontaneous rate plus 0.15 times the spike dynamic range from spontaneous to maximum. When the criterion rate fell between the rates at two adjacent sound levels the threshold was calculated using linear interpolation. To de-emphasise any small random areas in the response area, and as a consistency check, it was required that the threshold criterion was also exceeded for 10 dB above threshold at that frequency.

A similar operation was used independently at each frequency to find the highest level at which the firing exceeded the threshold criterion. This traced out the upper edge of any circumscribed response areas.

CF was taken as the frequency at which the threshold criterion was fulfilled at the lowest sound level. Frequency bandwidths at 10 and 40 dB above minimum threshold were taken as the most extreme low and high (interpolated) frequencies where the traced out tuning curve crossed these levels. *Q*_10_ and *Q*_40_ were calculated by dividing the CF by these two bandwidths.

The equivalent rectangular bandwidth (ERB; Patterson, 1976; Moore & Glasberg, 1983) was also calculated from the threshold tuning curve. This was achieved by integrating across the lower edge of the entire receptive field (ignoring any non-monotonicity in the tuning curve), to calculate the total power that would pass through a linear filter of this shape. The equivalent rectangular filter was calculated that would pass the same power, and the width of this filter calculated to give the ERB.

To ensure the reliability of the calculated values the analysis was repeated with different degrees of smoothing across frequency (0.04, 1/16, 1/8, 3/16 octaves) and the values rejected from further analysis if they were inconsistent. If the values were consistent across smoothing and with the audio-visual estimates then the CF and threshold at CF were adopted from the 1/16th octave smoothing. If there was a disparity between the automatic and audio-visual estimates, then the response areas were viewed and a decision made whether to accept the automatic estimates, or whether to reject them as unreliable. It should be noted that although smoothing was used it was performed on up-sampled data; its primary purpose was to aid interpolation onto a consistent frequency grid and had little effect on the measured tuning widths as verified by the reliability check.

## Results

### Frequency response area classes in the IC

#### Subjective classification of frequency response areas

CFs of the units in our sample ranged between 0.041 and 35 kHz. Visual inspection of the 2826 response areas revealed several readily identifiable shapes within the population and we used these subjectively to define seven distinct response area types (Fig. 1): (1) V, the classic V-shape found for auditory nerve fibres (*n*= 1209); (2) VN, V-shaped but non-monotonic at CF (*n*= 553); (3) N (narrow), like V but with less frequency expansion at high levels (*n*= 138); (4) C (closed), completely circumscribed with no response at high sound levels to any frequency (*n*= 169); (5) TD (tilt down, *n*= 412) and (6) TU (tilt up, *n*= 87) have a region of excitation sloping upwards from CF towards low (TD) or high (TU) frequencies, respectively, leaving little response to CF tones at high levels; (7) D (double-peaked) having two low-threshold peaks (*n*= 18); and U, (unclassified, *n*= 240) not sufficiently reliable to allow classification or did not fit into any of the seven classes.

One important question is the disposition of these various classes of response area across the CF range of the animal. To address this issue Fig. 2 shows the subjective classes as a function of CF in octave bands extending across the animals’ hearing range. What is clear from Fig. 2 is that there are significant numbers of neurons exhibiting the different response areas across all frequencies. If we form a mean of all neurons of each response type across all CFs (Fig. 2, right column) the classes are preserved. Not surprisingly, if we form a mean of all response types at each CF (Fig. 2, lower row) the result is V-shaped with some evidence of non-monotonicity at CF. Note that when plotted on an ERB-rate scale the decrease in tuning width as a function of CF is much less pronounced than when plotted on a logarithmic frequency scale.

#### Principal components analysis of frequency response areas

**Figure 3:**
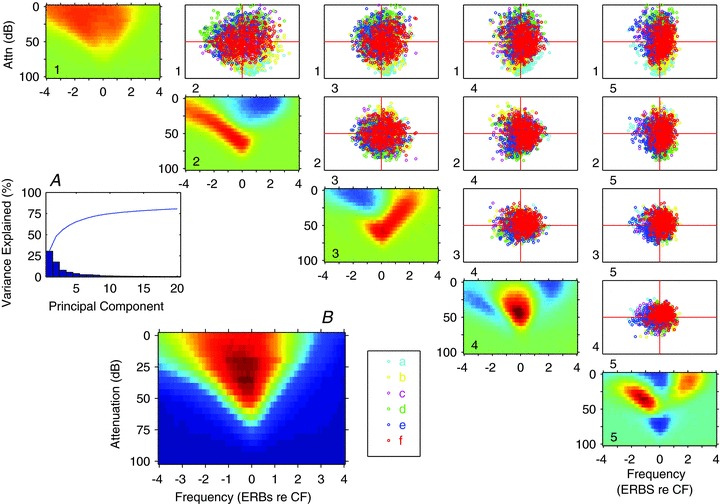
Principal component analysis of the frequency response areas *A*, bar plot of the variance explained by each of the first 20 principal components, the blue line shows the cumulative total. *B*, mean FRA obtained by normalising and summing all the FRAs. 1-5 (diagonally): the derived principal components. The scatter plots show each FRA in the sample, colour coded by its class assigned by clustering on the PCA weightings (see 6 clusters in Fig. 4), plotted according to the contribution of each pair of principal components to that FRA. For each plot the ordinate corresponds to the component to the left of each row, and the abscissa corresponds to the component shown at the base of the column. The plots below the diagonal would be mirror images so are not shown. The classes were obtained by *k*-means clustering of the 22 significant PCs.

To seek evidence of distinct clusters in IC response areas, we analysed receptive fields using principal component analysis (PCA), with response areas plotted on an ERB scale. This has the advantage that it contains no *a priori* assumptions about which features were important. Figure 3*A* shows the variance explained by each of the principal components (bars) together with the cumulative variance (continuous line). About 70% of the variance is explained by the first five components, with components 6 and higher contributing relatively little to the total.

Figure 3*B* shows the mean frequency response area obtained by summing all of the normalised response areas. The smaller frequency response areas in the remainder of the figure represent the first five principal components (PCs) which, when added in appropriate proportions to the mean frequency response area, generate the whole range of frequency response area shapes in the sample. Interestingly, even some of the individual PCs map closely to the different subjective classes of response area and their associated inhibitory areas. For example, the second component closely matches the form of TD response areas and the third component resembles the TU class while component 4 reflects C-type units.

**Figure 4:**
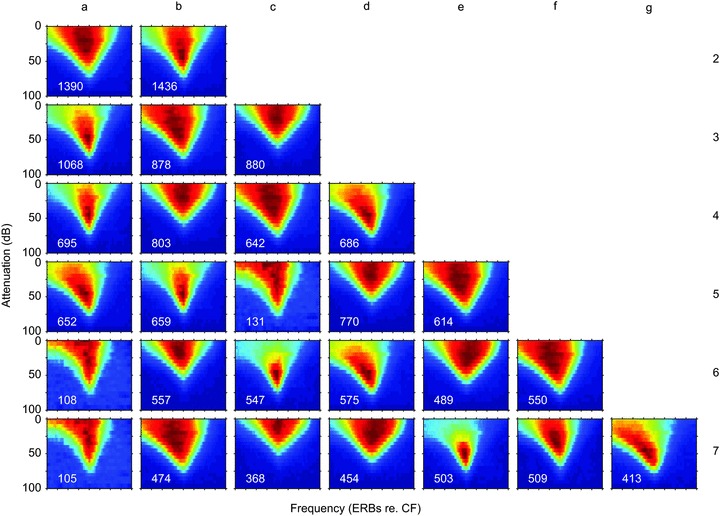
Classification of the response areas by PCA followed by cluster analysis of the first 22 principal components of the response area, as shown in **Fig. 3** The clusters have been computed for 2–7 clusters which we refer to by the letters a–g to distinguish them from clusters and classes in the rest of the paper.

Parallel analysis (Horne, 1965) was used to determine that the first 22 principal components were significant and these were used as the input to a *k*-means clustering algorithm, allowing the number of clusters to vary between 2 and 7. The results are shown in Fig. 4 with the clusters labelled a–g to distinguish them from other clusterings. The clustering method produces the clusters in random order, so there is no correspondence vertically down a column. It is clear that the clustering mainly produces V-like response areas of varying threshold, monotonicity and width. Only with 3–5 clusters does anything resembling an N-type emerge. For five clusters and above the method produces several V-like clusters, a TD-like and a C-like cluster. All other extracted classes are more or less V-shaped and the nuanced distinctions between V, VN, TU and N are not resolved by this clustering based upon the 22 PCs.

Each of the scatter plots within the matrix in Fig. 3 shows the position of the response types based on pairs of components representing all combinations (1 *vs.* 2; 1 *vs.* 3; 2 *vs.* 3 etc.) within the two-dimensional space of the principal components. There is no clear separation of the different coloured points representing the response classes in the scatter plots, nevertheless the clusters are not completely overlapping, so there are regions where one class dominates. Any three components could also be plotted in a three-dimensional plot that could be rotated to allow the cloud to be viewed from any angle, but this failed to reveal any further evidence of segregation between the various classes. Thus, this analysis demonstrates that although different response types can be generated by adding the appropriate PCAs in the right proportions, there is no clear separation of the different types.

**Figure 5:**
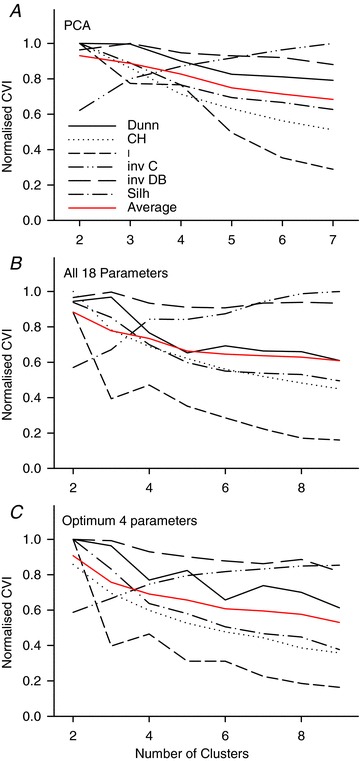
Cluster validity indices (CVIs) as a function of the number of clusters The various indices indicate the quality of the clustering. For data falling naturally into a specific number of separable clusters the indices would give an index with a maximum for that number of clusters. Values are normalised to the maximum in the range. *A*, CVIs for the PCA-based cluster analysis shown in Figs 3 and 4. *B*, CVIs for clusters based upon all 18 parameters shown in Fig. 7. *C*, CVIs for clusters based upon the optimum set of parameters for each number of clusters shown in Fig. 8. *B* and *C* are normalised to the same maximum, so are directly comparable. Dunn, Dunn index (Dunn, 1973); CH, Caliński–Harabasz index (Caliński & Harabasz, 1974); I, I index (Maulik & Bandyopadhyay, 2002); inv C, inverted C index (Hubert & Levin, 1976); inv DB, inverted Davies–Bouldin index (Davies & Bouldin, 1979); Silh: silhouette (Rousseeuw, 1987); red curve, mean of CVIs after normalisation.

The quality of clustering can be measured by CVIs. The six CVIs that result from the PCA clustering, normalised to the maximum of each index, are shown in Fig. 5*A*, along with their mean taken after normalisation. With one exception (the inverted C index) the CVIs tend to reduce as the number of clusters increases, showing that the best clustering occurs with the lowest number of clusters. This again demonstrates that clustering based upon the PCAs achieves poor segregation.

#### Parameter-based cluster analysis of frequency response areas

**Figure 6:**
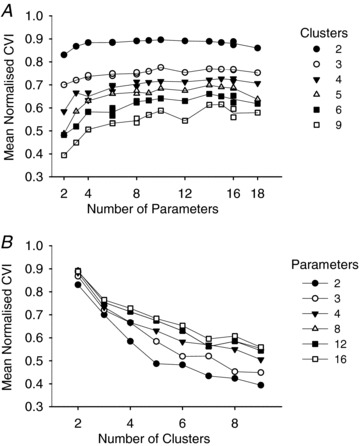
Mean CVI as a function of the number of parameters used and the number of clusters requested *A*, number of clusters as the parameter. *B*, number of extracted parameters as the parameter.

Deriving the PCAs and the subsequent attempt at clustering used all the information available in the FRA. However, it is possible that irrelevant information in the raw PCAs diluted the impact of the more relevant information. We therefore extracted 18 parameters from the FRAs (Table 1) that characterised them across frequency and level. These parameters were likely to be highly correlated with each other, so we adopted an automatic protocol to find the optimum subsets of parameters, varying systematically the number of parameters chosen and the number of clusters required (see Methods). The mean CVIs obtained by this process are shown in Fig. 6. Different orders of parameter selection could result in the same number of parameters, so there are multiple points closely overlying from four clusters upwards. From Fig. 6*A* it can be seen that there is a broad maximum in the mean CVI between 4 and 18 parameters with little or no change up to the full set of 18. Figure 6*B* demonstrates that clustering is most distinct (highest CVIs) with only two clusters, declining markedly for larger numbers of clusters. It should be stressed that these calculations used the best possible combinations of parameters for each point, so the highest degree of clustering possible is indicated by each point. This again demonstrates that the data do not fall into distinct clusters even when the parameters are specifically chosen to maximise this outcome.

**Figure 7:**
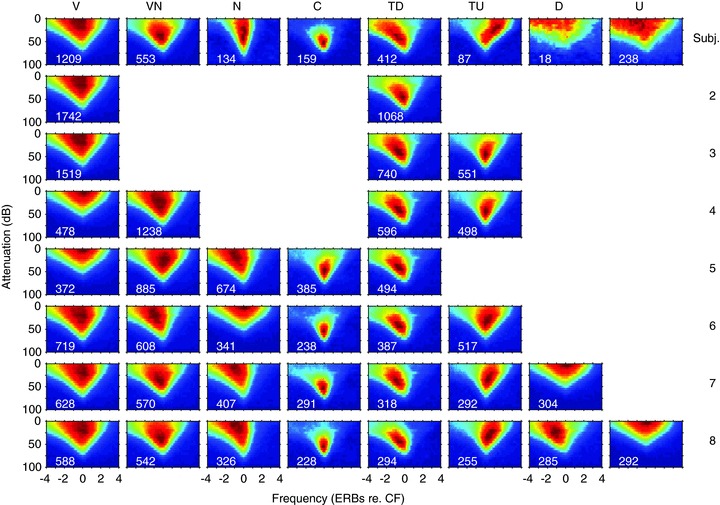
Mean frequency response areas of the clusters obtained using *k*-means clustering as the number of clusters is varied using all 18 extracted parameters The subjective classes are shown in the top row and the number of clusters is shown on the right.

**Figure 8:**
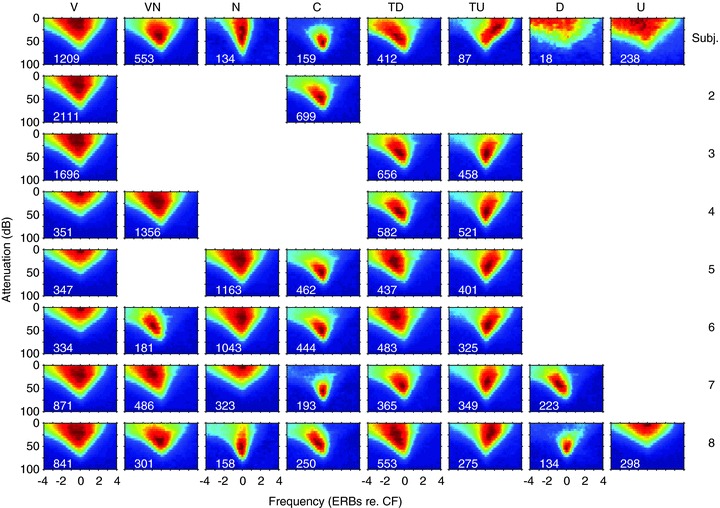
Mean frequency response areas of the clusters obtained using *k*-means clustering as the number of clusters is increased using only 4 extracted parameters that were optimised separately for the number of clusters Format as in Fig. 7.

Given the very broad maximum (from 4–18 parameters) in Fig. 6*A* we computed the clustering based upon the two extremes (4 and 18). The clusters obtained are shown as a function of the number of clusters allowed and the frequency in Figs 7 and 8 in which the top line also shows the subjective classes. The row position of the clusters was determined by a distance metric comparing the mean parameter values for the units in the subjective classes with those in the clusters. This resulted in similar-shaped FRAs lining up vertically. When all 18 parameters were used (Fig. 7) the two cluster conditions returned the, almost inevitable, V-shape and a shape resembling the TD response area. For three clusters the shapes were V, TD and a shape that appeared to be an amalgam of TU, C and N. By six clusters nearly all of the single peaked subjective classes could be identified with the notable exception of anything resembling the N class. With more than six clusters the new clusters were not qualitatively different from those that had emerged previously, but merely further variations in the V and VN classes.

The clustering based upon only four parameters is shown in Fig. 8. Note that the optimum set of four parameters was chosen individually for each number of clusters and therefore they are not necessarily the same parameters for every row. It is clear that using only four parameters in this way achieves almost the same result as using the full 18 parameters. However, the six clusters obtained with four parameters appear to be less distinct and clearly separable than when using 18; compare, for example, the C type for six clusters in Figs 7 and 8.

The absence of an N class was a feature of all our manipulations and suggested that the PCAs and the parameters we extracted, after correction for tuning variation with frequency, were insufficiently sensitive to the narrower response area at high sound levels that distinguishes the N class. Nevertheless, rather than make another arbitrary distinction we have labelled the cluster with parameter values least dissimilar from the subjective N class as 3(N).

Interestingly the D class of response area was not identified by any of our objective measures. This is probably because D-type units were very rare in the sample (18/2826) and the parameter space was designed to emphasise differences between single peaked response areas.

**Figure 9:**
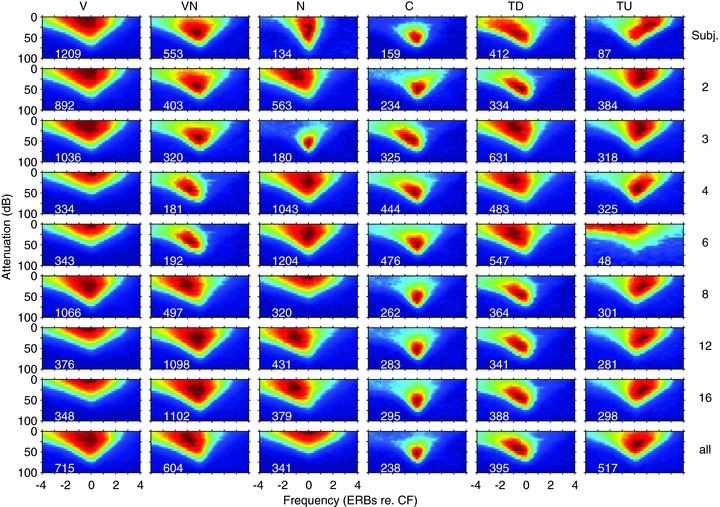
Mean frequency response areas of the clusters obtained using *k*-means clustering as the number of clusters is fixed at 6 and the number of parameters included in the clustering is progressively increased The format is as in Fig. 7 with the number of parameters included listed to the right.

Finally, given that with six clusters we were able to identify most of our subjective classes, we computed the six clusters using different numbers of parameters in case any specific parameter combination achieved significantly better clustering (Fig. 9). What is clear from this figure is that V, VN, C and TD classes emerge irrespective of the number of parameters, while N and D never appear.

The quality of the clustering obtained using the 4 and 18 parameters can be seen from the remaining two plots of CVIs in Fig. 5. Whether we used all 18 (Fig. 5*B*) or only the optimum 4 parameters (Fig. 5*C*), the maximum mean CVI occurred at 2 clusters. However, the decline from this maximum with up to 8 clusters was not precipitous for most of the indices.

**Figure 10:**
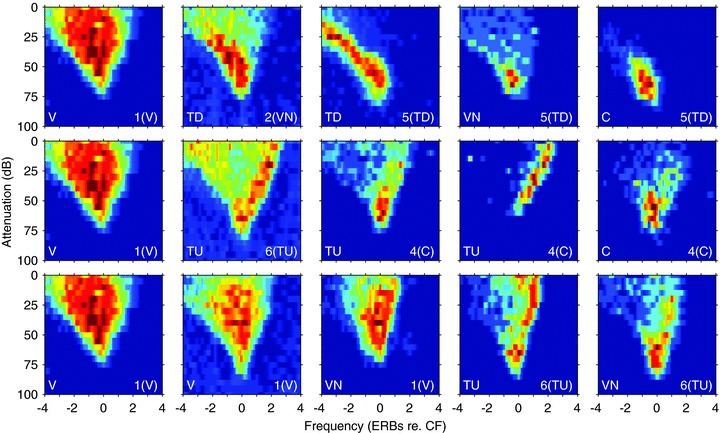
Selected frequency response areas with approximately similar CFs (1.03–2.23 kHz), showing progressive changes in characteristics across a continuum The same V-shaped unit is shown in all three rows The label to the bottom left of each plot is the class determined subjectively and the label to the right is the objective cluster class using all 18 parameters.

Taken together these findings lead us to conclude that although subjective classification can identify exemplars of distinct classes of response area (Fig. 1), these represent the end-points of continua rather than discrete clusters. We have illustrated this in Fig. 10 by selecting sequences of FRAs that appear to reflect transitions between V-shaped and other classes. What is also clear from this figure is that units with features that appeared obvious to visual inspection and hence led to classification (e.g. the fifth FRA in the top row) appear in other classes after the objective process probably because the parameterisation extracts features which are not the same as those used subjectively. Perhaps most striking in this respect is the failure of the objective protocol to classify the fourth FRA in the second row as a TU. This is likely to be because the extreme non-monotonicity at CF overrides the presence of a response above CF.

### Quantitative analysis of frequency response area properties

#### Distribution of unit thresholds as a function of characteristic frequency

The thresholds of the most sensitive units in the sample lie close to the behavioural threshold (Prosen *et al.* 1978). There is a greater discrepancy between the behavioural and unit thresholds at frequencies above ∼3 kHz. This is probably due to differences in the experimental protocols used to gather single unit and behavioural data. The latter employs free-field stimulation allowing for modifications to the sound field by the pinna, concha and external meatus, whereas closed-field presentation in the physiological experiments eliminates these factors. For the majority of the data, the spread of thresholds at any frequency is less than 30 dB, but some unit thresholds are higher than the lowest at that frequency by >40 dB. There was no obvious tendency for any of the response types to have consistently higher or lower threshold than the others.

#### Estimates of response area bandwidth

Obtaining representative values of frequency bandwidth and tuning for units in which the width of the frequency response area changes with level is problematic. For auditory nerve fibres two estimates of tuning have been derived, *Q* (the ratio of the CF to the bandwidth at some level, typically 10 dB, above threshold), and the ERB (which estimates the tuning of the unit in terms of a rectangular filter that would pass the same power as the empirically measured filter shape). The difficulty in estimating bandwidth is compounded for units in the IC because, while some are V-shaped, other classes have a much more irregular form and may not be symmetrical. We calculated the frequency bandwidth of units at 10 dB and, where the response area permitted, 40 dB above threshold. The bandwidth at 40 dB is interesting because, as is evident from the units illustrated in Figs 1 and 2, the most obvious differences between the response area classes occur at the higher sound levels. These bandwidth measures were used to calculate both *Q* (at 10 and 40 dB above threshold) and the ERB to facilitate comparison with previously published measurements at other levels of the auditory pathway and in other species.

**Figure 11:**
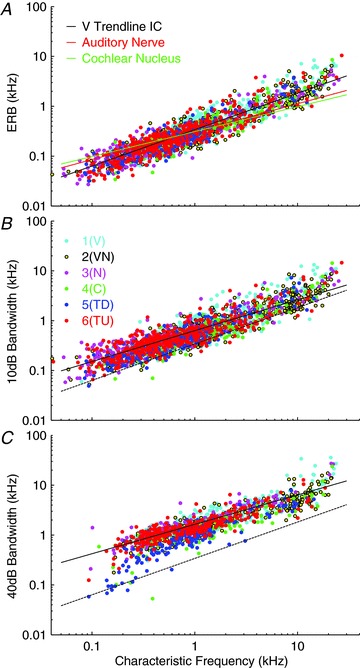
Equivalent rectangular bandwidth (ERB) (*A*), and bandwidth at 10 dB (*B*) and 40 dB (*C*) above threshold as a function of characteristic frequency *A*, lines show ERB estimates from auditory nerve and cochlear nucleus from Evans *et al.* (1992; red line) and Sayles & Winter (2010; green line) and a trend line fitted to the V-type data for easy comparison (black line). *B*, the continuous line shows the trend line fitted to the V-type data; the dotted line shows the trend line from the ERB data repeated from panel *A*. *C*, continuous line shows the trend line fitted to the V-type data; the dotted line shows the trend line from the ERB data repeated from panel *A*. Unit classes derived from the clustering based on all 18 parameters with 6 clusters (cf. Fig. 7) are indicated in the key: cyan, 1(V); yellow, 2(VN); magenta, 3(N); green, 4(N); blue, 5(TD); red, 6(TU).

Figure 11*A* shows the variation of the ERB computed from the threshold tuning curve as a function of characteristic frequency. For a Gaussian curve the ERB is equivalent to the half-power or 3 dB bandwidth and is approximately 46% of the 10 dB bandwidth, and this is approximately true for the data here. All measurements based on simply measuring a single point on the threshold function are prone to error and variability, but the ERB measure is less so, as indicated by the tight spread of the computed bandwidths. The slope of the line fitted to the V-type units is 0.73 (ERB = 0.34 × CF^0.73^). These ERB values do not take into account the non-monotonicity in the response areas of many IC units, but since most of the power passes through the filter at frequencies close to the tip of the tuning curve, where the unit is most sensitive, the values do give a reliable indication of the tuning in the tip region. The red and green lines in Fig. 11*A* are the equivalent measurements for cochlear nerve fibres and cochlear nucleus units in the guinea pig reported by Evans *et al.* (1992) and Sayles & Winter (2010), respectively, and show that the slope of the relationship between ERB and CF for all these groups of units is strikingly similar. If a number of high-frequency units rejected by Evans *et al.* (1992) from his calculation are included then the auditory nerve fit is more similar to our IC fit.

**Figure 12:**
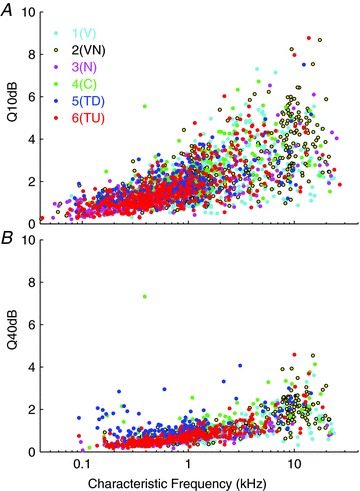
*Q*_10_ (*A*) and *Q*_40_ (*B*) values as a function of characteristic frequency Six unit classes derived from the 18 extracted parameter clustering are indicated in the legend and are as in Figs 7 and 11.

**Figure 13:**
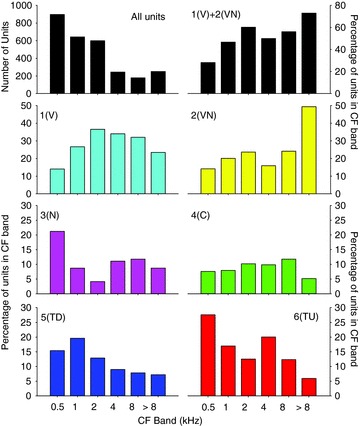
Distributions of the CFs of the six response classes derived from the 18 extracted parameter clustering The distribution of the CFs of the whole sample population is shown by the upper left bar plot. The remaining plots show the percentage of the units in each frequency band for each of the different frequency response classes. Classes are as shown in Figs 7 and 11.

**Figure 15:**
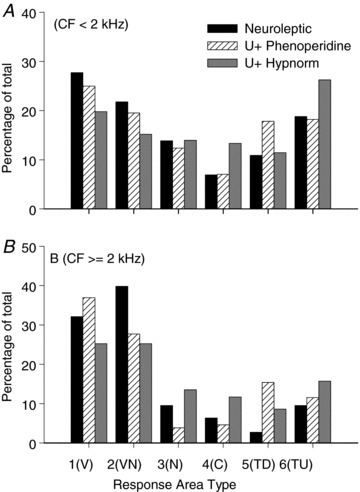
The influence of anaesthetic on the proportion of responses in the six classes derived by *k*-means clustering from 18 extracted parameters Classes are as shown in Figs 7 and 11.

Note that in Figs 11, 12, 13 and 15 the classes we plot were derived from the objective clustering using all 18 parameters (as shown in Fig. 7). We computed and replotted all four of these figures using the four parameter fit and using the subjective classes and, at least by visual inspection, could see no differences between the values extracted using any of these methods in terms of the degree to which the classes stand out in any of the plots.

The frequency bandwidths at 10 dB are plotted as a function of CF in Fig. 11*B*. As with the auditory nerve data (Evans, 1972), the bandwidth (BW) increases as a function of CF and the relationship is well fitted by a straight line on the log–log plot with a slope of 0.62 (BW_10_= 0.63 × CF^0.62^: fitted to the V units). Data from different response area classes cluster tightly around this line demonstrating that close to threshold there is little difference between the tuning for different classes of units.

Bandwidths measured at 40 dB above threshold are shown in Fig. 11*C*. The slope of the line of best fit (0.59; BW_40_= 1.64 × CF^0.59^: fitted to the V units) is shallower than the equivalent at 10 dB. It was not possible to measure a bandwidth for all units because many did not respond at 40 dB above threshold. This is most obviously the case for the highly non-monotonic 4(C) units and relatively few units of this type occur in Fig. 11*C*.

In general, values for response area bandwidths at 40 dB above threshold are more heterogeneous than those at 10 dB, and, at this higher level, the bandwidths of some classes of units differ from others. Perhaps surprisingly, although 4(C) (green dots) can be seen at the lower edge of the cloud of points at frequencies above 1 kHz they hardly stand out. This is likely to be because many 4(C) units did not yield values at 40 dB suprathreshold. It is important to remember that for both classes of tilt-like units (5(TD) and 6(TU)) the bandwidth measured at 40 dB will extend across a different frequency range to that at 10, but these are still plotted at their CF position. Given the removal of much of the excitatory response area at CF by putative inhibitory inputs TU- and TD-type units are likely to have suprathreshold bandwidths that are generally narrower than for V-types and it is clear that the blue dots representing the 5(TD) response areas have narrower 40 dB bandwidths, at least below 1 kHz, than other types in Fig. 11*C*.

Figure 12*A* represents the *Q*_10_ values calculated for the sample, plotted as a function of CF. As at other levels of the pathway *Q*_10_ increases with CF and the data become more dispersed at CFs above 1 kHz. While the data for most of the classes are interspersed, not surprisingly the same trends are apparent in this representation of tuning width. Class 4(C)-type units are generally found on the upper side of the distribution at all CFs, and these units display some of the highest *Q* values in the sample. Certainly, near threshold no class of units appears to be markedly more sharply tuned than other units with the same CF.

Figure 12*B* shows a similar plot for the *Q*_40_ values. Again not surprisingly, for nearly all classes the response areas are much broader at this higher level as reflected in their low *Q* values. The class 5(TD) and 4(C) units generally have higher *Q*_40_ values than the class 1(V). The few class 4(C) units that gave a measureable bandwidth at this level stand out as being sharply tuned because the measure cuts across at a level where the closure of the response area is nearly complete. At low CFs the class 5(TD) units also appear to be sharply tuned, presumably as much of their response area has been cut away by encroaching inhibition from higher frequencies.

#### Distribution of response classes with CF

A key question is the extent to which particular response types are distributed with respect to CF. To compensate for the fact that the data set was not evenly distributed across frequency (see first panel of Fig. 13), the number of units of each type was plotted as the percentage of the total number of units in CF octave bands from 0.5 to 8 kHz and everything above 8 kHz (see remaining panels, Fig. 13). A χ^2^ test of independence showed there was a significant difference in the distribution of response types across frequency bands (χ^2^ (25, *n*= 2810) = 426.26, *P* < 0.001). Nevertheless, although there are trends across frequency it is remarkable that all classes are represented in all frequency bands. The distribution of the subjective frequency response area classes with CF is shown in Fig. 2.

### Inhibitory areas revealed by the use of a second tone at CF

The shapes of the response areas and the evidence for continua between classes are consistent with inhibition playing an important role in shaping IC response areas as suggested by previous studies (Vater *et al.* 1992; Yang *et al.* 1992; Palombi & Caspary, 1996; LeBeau *et al.* 2001). To uncover possible areas of inhibition we measured response areas in the presence of a simultaneously presented CF tone at 10 dB above threshold in 198 units. Figure 14 shows the mean single-tone frequency response areas (in a similar format to those in Fig. 2) for units for which we also measured two-tone response areas (*n*= 198). Comparing Fig. 2 with the upper panel of Fig. 14 indicates that these units are a representative subset of our total sample. The lower part of Fig. 14 shows the average two-tone response areas for the same sample. The higher discharge rates at frequencies outside the single tone response area are due to the CF tone. Against this higher discharge rate areas in which the driven rate to the CF tone was reduced (dark blue) are clearly evident.

In class 1(V) units upper and or lower frequency inhibitory regions are apparent in many cases. The plots for class 5(TD) and 6(TU) units exhibit precisely the asymmetrical inhibitory areas that one would expect: high-frequency inhibitory sidebands for class 5(TD) and low-frequency for class 6(TU). The island of excitation in class 4(C) units results from inhibition that extends strongly across the whole frequency range and is only overcome by excitation near CF at levels within a few tens of decibels of the minimum threshold.

### Influence of anaesthetic type

As detailed in the Methods, several different anaesthetic protocols were used in these experiments that differed both in the drug agents and doses used. To determine whether there was any substantial difference between the response area types encountered under the different anaesthetic regimes we compared the distributions for three of the protocols which contributed the largest numbers to the complete sample: (1) neuroleptic, (2) urethane/phenoperidene and (3) urethane/Hypnorm (see Methods for details). To take into account differences in the numbers of units at low and high frequencies we plotted the percentage of each response area type for units with CFs below 2 kHz, and separately for those with CFs of 2 kHz or above (Fig. 15*A* and *B*). Although the distributions look quite similar, a χ^2^ test of independence showed there was a significant difference in the distribution of response types with anaesthetic regime for CFs <2 kHz (χ^2^ (10, *n*= 2101) = 47.90, *P* < 0.001) and ≥2 kHz (χ^2^ (10, *n*= 676) = 48.93, *P* < 0.001). However, with such large sample sizes even small differences in distribution may be statistically significant: the key point for this analysis is that none of the response types was restricted to a particular anaesthetic regime.

While the distributions are not identical for each of these anaesthetics all the response area types were observed under all the different anaesthetic regimes in both CF regions.

## Discussion

Our results show three main findings: (1) although frequency response areas of IC neurons can be assigned to one of several classes, these classes are not discrete but end points of a series of continua, (2) the different classes are represented across the frequency range, and (3) the shapes of response areas reflect the operation of inhibition. These conclusions are supported by the visual analysis of the response areas and two objective methods: cluster analysis of a set of extracted feature vectors, and clustering of the significant principal components of the response areas. Using subjective classification we recognised seven response classes, five of which were clearly recognised by the feature-based classification. The N-type was less well recognised in the objective analysis, while a seventh class, the double-peaked response area, was not found, probably because they were few in number, and the analyses were optimised for single frequency sensitivities. Importantly, many response areas had forms intermediate to the class exemplars, demonstrating that the classes did not form isolated clusters. Furthermore, we failed to observe separated clusters in the PCA. Two-tone response areas revealed patterns of inhibition consistent with the notion that inhibition determines the shapes of many IC frequency response areas by modifying the V-shaped tuning established in the cochlea. These continua and frequency distributions have important implications for understanding the origin of response areas in the IC and they point to an important role for the IC in afferent integration.

### Comparison with previous studies

The classes we identify are not frequency, species or anaesthetic dependent. They have all been observed previously in the auditory midbrain of other animals including birds, bats, rodents, carnivores and primates, under different anaesthetics or without anaesthesia, and independently of whether stimuli were presented contralaterally or bilaterally (Rose *et al.* 1963; Aitkin *et al.* 1975; Ryan & Miller, 1978; Ehret & Merzenich, 1988; Casseday & Covey, 1992; Yang *et al.* 1992; Palombi & Caspary, 1996; Ramachandran *et al.* 1999; Egorova *et al.* 2001; LeBeau *et al.* 2001; Hernández *et al.* 2005; Alkhatib *et al.* 2006; Schumacher *et al.* 2011). The three most consistently reported types are those we have termed V, N and C; however, TD, TU and D response areas have also been reported by others (Ehret & Merzenich, 1988; Egorova *et al.* 2001; LeBeau *et al.* 2001; Hernández *et al.* 2005; Alkhatib *et al.* 2006). We did not distinguish receptive field types containing multiple excitatory and inhibitory domains as reported in mouse (Egorova *et al.* 2001) and rat (Hernández *et al.* 2005). Across these different studies there is considerable variation in the proportions with which the different classes occur, probably a result of sample size and classification criteria. In our data, all response types were encountered with three different anaesthetic regimes (Fig. 15).

#### Response area types in IC

Responses areas can be divided into two broad categories: the most common are V-shaped response areas (V and VN), with the remainder (C, N, TD, TU) collectively termed non-V. V response areas resemble those recorded at the most peripheral level of the auditory pathway, the cochlear nerve (Kiang *et al.* 1965; Evans, 1972). In contrast, non-V-shaped response areas clearly show evidence of modification by central processing.

Frequency tuning data for IC neurons of all classes (Figs 11 and 12), as estimated by *Q*_10_, and the ERB, are like those measured in the guinea pig auditory nerve and cochlear nucleus, and show a similar increase with CF (Evans, 1972; Sayles & Winter, 2010). However, the fact that values are similar for V and non-V classes, despite their very different shapes, emphasises that the differences between classes are determined by their responses at supra-threshold sound levels; near threshold there is no significant enhancement of frequency tuning at the IC compared with the auditory nerve. Accompanying clear qualitative differences in monotonicity and the tilts in frequency with level in the non-V classes, *Q*_40_ values show some tendency to differ across classes, but considerable overlap remains.

#### The contribution of inhibition

Although V-shaped excitatory response areas appear very similar to auditory nerve responses, in response areas measured with a two-tone protocol evidence for inhibitory sidebands was apparent on either side of the excitatory region in some V-type response areas. Such a pattern could be obtained by sideband inhibition or by a V-shaped inhibitory response centred on CF that extends beyond the excitatory area. Similar observations have been reported in previous studies (Ehret & Merzenich, 1988; Egorova *et al.* 2001; Alkhatib *et al.* 2006). Experiments where GABA- and or glycinergic inhibition are blocked using microiontophoresis of receptor antagonists show that inhibition operating in the IC, although influencing their firing rate, does not usually shape the excitatory region of most *V-type* units (Palombi & Caspary, 1996; LeBeau *et al.* 2001; Davis, 2005).

In contrast, the excitatory regions of non-V response areas are shaped by inhibition to varying degrees (Fig. 14), which probably accounts for the failure of our analyses to isolate discrete clusters of response areas. Two-tone response areas for N and C units show their excitatory response areas are often accompanied by pronounced areas of inhibition at frequencies above and below the excitatory region (Fig. 14). The frequency range of this inhibition can be extensive, extending two or more octaves either side of the neuron's CF (Egorova *et al.* 2001) and in some cases inhibition encroaches over the top of the region of excitation giving rise to a highly non-monotonic response. Variation in the strength of this inhibition leads to response areas intermediate between the N and C types. Marked areas of inhibition are also a feature of the TU and TD response areas (Fig. 14). In these classes, it dominates at frequencies above or below the CF, consistent with a partially overlapping inhibitory input from neurons with CFs higher or lower than the recorded unit. Depending on the degree of overlap, the interaction could lead to a V-shaped excitatory response area becoming tilted or closed (Figs 10 and 14). Such varying patterns of inhibitory input almost certainly underlie the continua of response area shapes apparent in our data.

The two-tone protocol we applied does not demonstrate that the observed inhibitory activity necessarily resides within the IC, but the effects observed in studies that used microiontophoresis of antagonists to GABA and glycine in the IC demonstrates that at least in some cases it does (Vater *et al.* 1992; Yang *et al.* 1992; LeBeau *et al.* 1996; Palombi & Caspary, 1996; LeBeau *et al.* 2001). Collectively, these iontophoretic studies show that inhibition in the IC modulates the firing rates of most neurons, and in the case of units in the non-V classes it has a profound influence in shaping the excitatory component of the response area.

As a caveat, two-tone response areas include a contribution from two-tone suppression generated on the basilar membrane. However, the major contribution is likely to be from inhibition, since similar inhibitory patterns are obtained with single tones when spontaneous activity is present (Egorova *et al.* 2001; Alkhatib *et al.* 2006), and the patterns of inhibition differ from those for two-tone suppression in the auditory nerve (Sachs & Kiang, 1968).

#### Inheritance of response area properties in the IC?

The presence of continuous distributions of response properties argues against the notion that response types reflect relatively specific lines of afferent input from individual nuclei in the brainstem. Ramachandran *et al.* (1999), reporting data from the IC of decerebrate cat, identified three distinct types of response areas: types V, I and O (corresponding to our V, N and C classes, respectively). On the basis of the distribution of these types across frequency and on their binaural and other properties, they proposed that each type reflected one of three sources of brainstem input: type V, restricted to low frequencies, from the medial superior olive; type I from the lateral superior olive; and type O from the DCN (Davis *et al.* 1999, 2003; Ramachandran *et al.* 1999; Davis, 2002; Ramachandran & May, 2002). In contrast, we observe a larger number of response types, and all types occur over the whole range of CFs. Other studies, including one in an unanaesthetised preparation, similarly report V-type response areas over the entire frequency range (Egorova *et al.* 2001; Hernández *et al.* 2005; Alkhatib *et al.* 2006). Additionally, IC response areas include the TD and TU classes that are not commonly reported in brainstem recordings. It is not clear if the findings of Ramachandran *et al.* (1999) represent a real difference between cat and other species, or can be attributed to differences in experimental method (Davis, 2005). In any event, our findings point to the importance of excitatory and inhibitory integration in the formation of IC responses areas.

We began this study with a subjective classification as traditionally used in very many auditory papers. It was clear that some units were very easy to classify, but a larger number were more difficult, because they exhibited aspects of different classes to different degrees. We attempted to mechanise the process using a variety of automated techniques aimed at minimising bias; some of these are reported in this paper, others, including the creation of a decision tree (cf. Blackburn & Sachs, 1989) were rejected. What has emerged from our attempts at objective classification is that although subjectively it is easy to find extreme examples and use these to form the basis of classes, the vast majority of units lie between these extremes without forming discrete clusters.

In conclusion, the continua and frequency distributions we report would not be expected if the frequency response properties of an individual IC neuron were simply inherited from one of three brainstem nuclei. Rather, these data support a multi-staged integration of response properties both along the up-stream auditory pathway, and importantly within the IC itself.

## Abbreviations

BF: best frequency
C: closed
CF: characteristic frequency
CVI: cluster validity index
D: double-peaked
DCN: dorsal cochlear nucleus
ERB: equivalent rectangular bandwidth
FRA: frequency response area
IC: inferior colliculus
N: narrow
PC: principal component
PCA: principal components analysis
TD: tilt down
TU: tilt up
*Q*: quality factor
V: V-shaped
VN: non-monotonic V-shaped
SPL: Sound Pressure level (dB re. 20 micro Pascals)
